# Comparative Evaluation of Pharmacy Students’ Knowledge and Skills in Maternal and Child Health: Traditional versus Integrated Curriculum

**DOI:** 10.3390/pharmacy10030062

**Published:** 2022-06-07

**Authors:** Elizabeth Oyebola Egieyeh, Angeni Bheekie, Mea van Huyssteen, Renier Coetzee

**Affiliations:** 1 Discipline of Pharmacology and Clinical Pharmacy, School of Pharmacy, University of the Western Cape, Private Bag X17, Bellville, Cape Town 7535, South Africa; abheekie@uwc.ac.za (A.B.); mvanhuyssteen@uwc.ac.za (M.v.H.); 2 School of Public Health, University of the Western Cape, Private Bag X17, Bellville, Cape Town 7535, South Africa; recoetzee@uwc.ac.za

**Keywords:** curriculum, traditional, integrated, final year pharmacy students, maternal and child health, knowledge, skills, South Africa

## Abstract

Reducing maternal and child mortality is a health priority in South Africa. Therefore, health professional education should produce graduates that can meet these needs. This study compared the maternal and child health (MCH) knowledge and skills of cohorts of final-year students exposed to a traditional (in 2017 and 2018) and integrated (2019) curriculum using a 34-item questionnaire. Between the 2019 and 2017 cohorts, ANOVA and post hoc analysis showed significant differences in the reproductive and sexual health component which was dispersed in the second and final years of study (*p* = 0.007, Mean Difference (MD) = 8.3) andneonatal and child care (*p* = 0.000, MD = 15). while it was only in maternal and antenatal care (*p* = 0.009, MD = 10.0) for the 2019 and 2018 cohorts. Significant differences were observed in participants’ average mean scores (*p* = 0.000 for 2018 and 2017). The highest mean scores were recorded by the 2019 cohort in the three assessments. A one-sample t-test showed the highest mean differences in the reproductive and sexual health components (*p* = 0.000; MD 2017 = 12.4, MD 2018 = 14.8, MD 2019 = 20.7). Overall, the integrated MCH curriculum and the longitudinal dispersion of content enhanced students’ knowledge and skills.

## 1. Introduction

Like many sub-Saharan African countries, South Africa grapples with the burden of maternal and child mortality (MCM) [1]. To achieve the targets of the Sustainable Development Goal (SDG) 3 for the reduction of MCM, South Africa aims to half its current institutional maternal (120 per 100,000 live births in 2019) and neonatal mortality (25 deaths per 1000 live births in 2018) by 2030 [2].To accomplish this goal, a maternal, perinatal, and neonatal health (MPNH) policy that offers a framework for delivering valuable, all-inclusive, and cohesive maternal and neonatal services was developed [2]. The scope of the policy covers all interventions aimed at improving the health outcomes of individuals during the reproductive lifecycle. In addition, the policy intends to focus on all frontline workers to ensure universal health coverage through the National Health Insurance (NHI) plan.

Although pharmacists are not mentioned as part of the MCH workforce in the MPNH policy, their roles cover interventions in obstetric pharmacotherapy health promotion and disease prevention activities in contraception, preconception, pregnancy and infant care that target drivers of MCM similar to those outlined in the policy [3,4,5]. The provision of health promotion and preventive care is one of the goals of primary health care (PHC) as an antidote to some emergency obstetric care needs in MCH in South Africa [6]. Despite these potentials, community pharmacy operations are often disconnected from national population-based primary care programmes due to the fragmented health care system in South Africa [7].

Integrating pharmacy MCH services into the mainstream national MPNH policy would further enhance these contributions, foster accountability, and promote health system strengthening, which is a priority of the National Development Plan 2030 [8,9]. The need for integration has become urgent and demands that proven interventions be scaled up and accelerated since the onset of the COVID-19 pandemic [10]. The situation was emphasised in recent publications that projected a global reversal of the progress made to reduce MCM in the past three decades, which was attributed to the impact of the pandemic [10,11,12,13,14]. A South African Medical Research Council publication reported a double pandemic in the country due to the rise in adolescent pregnancy in this period [10]. One of the reasons for the rise was the lack of access to contraceptives at public PHC facilities during the hard lockdown. The author recommended that free contraceptives should be provided at schools, community settings, and community pharmacies where advocacy messages can also be promoted, such as in the health care facilities.

However, studies from several countries have shown that most practising pharmacists feel uncomfortable and ill-prepared to render MCH services [5,15,16]. Inadequate curriculum exposure, lack of relevant resources and continuing professional development in MCH were identified as some of the reasons for the ineptitude observed [5,16,17].

Like most discipline-based curricula, the traditional pharmacy curriculum is teacher-centred, non-contextualised and compartmentalised, leading to fragmented learning in silos and inadequately trained graduates [18,19]. In a study carried out at a medical school in Pakistan, one of the reasons hypothesised for the lack of reduction in the high rate of MCM was the fragmented MCH curriculum adopted at the school [20]. By introducing an integrated MCH curriculum through a team that cut across disciplines such as obstetrics and gynaecology, paediatrics, community medicine, pathology, and pharmacology, students’ knowledge, skills and ability to resolve difficult issues related to infant mortality were enhanced.

Curriculum integration involves the deliberate fusing or scaffolding of a premeditated educational experience either vertically or horizontally [19,21]. An integrated curriculum offers educational experiences that are coherent, contextual, engaging, nestled, connected, and dispersed longitudinally to motivate students and enhance learning, knowledge retention, and application [22]. In addition, it helps students to develop higher-order learning and problem-solving abilities [19,20]. In undergraduate MCH training, the effectiveness of curriculum integration is consolidated when linked with community-based learning and MCH PHC facility engagements, which foster preventive thinking skills and multisectoral collaboration [2,20,21].

For pharmacy to be socially accountable in MCH, the priority health needs of the nation should underpin undergraduate curricula. ‘Entry-level’ pharmacists should be knowledgeable and appropriately skilled to offer essential services [23,24]. The study follows two previously published studies. The first was an evaluation of the knowledge and skills of 2017 final-year pharmacy students that were exposed to a traditional, fragmented MCH curriculum [25]. The result obtained from the first study showed a knowledge and skills gap hypothesised to be associated with the traditional curriculum. Subsequently, a framework for an integrated MCH curriculum was developed and implemented [22]. The current paper compared the effect of the two exposures on students’ knowledge and skills for teaching and learning improvement. To our knowledge, the study is the only one at present that has compared a traditional and an integrated MCH curriculum in undergraduate pharmacy education in South Africa.

## 2. Materials and Methods

### 2.1. Study Design

The longitudinal, comparative evaluation study assessed the MCH knowledge and skills of three cohorts of final year (fourth year) undergraduate Bachelor of Pharmacy (BPharm IV) students from 2017 to 2019. The 2017 and 2018 cohorts were exposed to a traditional MCH curriculum in all four years of their study from 2014 and 2015 [25]. The 2019 cohort participated in the intervention to integrate the curriculum from the second (2017) to the final year of study. As such, the cohort had three exposures to the same questionnaire: pre and post intervention in 2017 (eight weeks apart) and two years later in the final year [22]. The MCH content of the curriculum was concentrated in the second year of study in both exposures [22,25].

### 2.2. The Traditional Curriculum

The content of the traditional MCH curriculum included lectures on pregnancy and infant care, communicable diseases, immunisation, and contraception. Due to timetable constraints, the pharmacology and clinical pharmacy discipline taught all other topics except for contraception in a 2 h crash course (Table 1). However, contraception lectures were taught by the pharmacy practice discipline and split into two parts that were longitudinally dispersed in the second (contraceptive methods in the National Standard Treatment Guidelines and Essential Medicines List (STGs and EML) at the PHC level) and final years of study (contraceptive methods not that are not available in the STGs and EML) [26]. The second-year lectures prepared the students for the Service-Learning in Pharmacy (SLiP) sessions at the MCH units of PHC facilities in the Cape Town metropole under the direct supervision of facility nurses [22,25]. Although students were exposed to these topics, the link between the topics and the continuum of care in MCH was not established. As such, students were completely ignorant of the broader concept of MCH; they only understood each topic in isolation, which led to compartmentalised and fragmented learning [25].

### 2.3. The Integrated Curriculum (The Intervention)

In response to government’s initiatives to reduce the MCM rate in the country, an intervention was introduced in 2017 at the second-year level to develop an integrated framework for MCH education at the school [22]. The framework aligned with three SAPC domains and the corresponding competencies (Table 1). Existing curriculum content with bearing to the continuum of care in MCH was identified at each year level, revised where necessary, and incorporated into the framework. An example is the communicable diseases lectures that were streamlined and contextualised to focus only on childhood communicable diseases. At the same time, emphasis was laid on the expanded programme on immunisation in South Africa in the immunisation lectures. In addition, new content such as preconception lecture and infant growth assessment practical were included in the framework at the second-year level, and an MCH externship (48 h training programme in a pharmacy or PHC facility undertaken during the mid-year holiday) component was introduced in the third year of study to complete the vertical integration.

To integrate the espoused, enacted, and experienced curriculum, an orientation session was organised in 2017 at the beginning of the second semester of the second year of study in the pharmacology and clinical pharmacy module (PHC223), where the main intervention was implemented [19,22]. The integrated framework used an instructional scaffolding design to promote competence in MCH knowledge and skills [27]. Students were informed of the components of the framework and the different year levels at which they would be exposed to each. The significance of the framework to pharmacists’ roles in MCH and the country’s high mortality and teenage pregnancy rate was highlighted during the orientation session. The link between each component (topic or activity) and the continuum of MCH care was established at the orientation session and during each contact session by the lecturer (primary author, EE) to ensure clarity and integration of content. Students were invited to participate in a longitudinal study two years later in their final year of study [22].

Different teaching methods such as infant growth assessment practical, contraceptive products demonstrations, didactic lectures, and experiential learning sessions (Table 1) at MCH units of PHC facilities under the direct supervision of facility nurses (for interprofessional and multisectoral collaboration) were included in the framework [2,22,28]. Students were encouraged to identify a gap in service delivery that they could fill at each health facility [29]. Assessments were carried out using different methods such as quizzes, tests, reflective writing, group case study, practical demonstrations, and final examinations within the allocated modules and in line with university regulations (Table 2). Other exposures to these topics horizontally or otherwise may have been through references to the importance of health education and medication safety in pregnancy and paediatrics offered during the other undergraduate course content in pharmaceutics, pharmaceutical chemistry, and pharmacotherapy lectures.

### 2.4. Study Participants

A total of 142 students participated in the study. The 2017 and 2018 cohorts had 54 and 41 participants, respectively, while the 2019 cohort had 47 participants. The criteria for participation in the 2019 assessment included participation in the 2017 MCH pre and post-intervention assessments and completion of the 2018 MCH component of the externship [22]. Participants who repeated third-year pharmacology and clinical pharmacy modules were excluded from the 2017 and 2018 assessments [25].

### 2.5. Study Questionnaire

The questionnaire was developed in 2017 (see the Supplementary Materials for the questionnaire) and used in the three studies. The structure and content were developed from different sources such as previous similar studies, the interventions outlined in the FIP statement of policy and the SAPC GPP guidelines for MCH [16,20,30,31,32]. The appropriateness, readability, clarity and length of the content of the 34-item questionnaire were assessed by four faculty versed in the subject, five final-year students recruited as assistant researchers and another eight final-year students who participated in the pilot test [25]. The pilot test was carried out once with one group of students in 2017. The questionnaire had three main Sections A–C. Section A contained questions on participants’ demographics, including locum experience (a person who substitutes for another person from the same profession to fulfil their duties temporarily) and parental status.

Section B had 28 items that assessed participants’ knowledge using three MCH components; B1 was reproductive and sexual health, which focused on contraception knowledge (9 items, 9 marks); B2 was maternal and antenatal care, which covered preconception and pregnancy care (10 items, 17 marks); B3 was neonatal and child care, which covered infant care, nutrition, childhood diseases, and immunisation (9 items, 12 marks). Section C was a written evaluation of participants’ infant growth assessment skills and knowledge (6 items, 8 marks). More information about the questionnaire development can be accessed from previously published articles on the study [22,25].

### 2.6. Recruitment and Data Collection

As much as possible, the same procedures were followed to recruit participants and collect data in the three assessments. Information about the study was provided to each class through emails sent by the primary author (EOE) using the university’s electronic communication platform. In addition, each year, six students who were allocated to the study as student researchers to meet course requirements for their final-year projects provided information and updates to the class. The class was informed that participation in the study was voluntary. Participant recruitment took place during a regular lecture period and at a designated venue where all final-year students were in attendance. Interested students were given the study information sheet and asked to complete the consent form before completing the self-administered questionnaire. Completion of the questionnaire mimicked the protocol required for a class test. The research assistants and the primary author served as invigilators. Completed questionnaires were returned to the researchers after 60 min [22,25]. Ethics approval was obtained from the Ethics Committee of the University of the Western Cape (HS/17/5/12, 24/07/2017; BM18/4/8, 08/06/2018).

### 2.7. Data Evaluation and Analysis

Trained student researchers graded and moderated the completed questionnaires using a structured memorandum. An independent research assistant captured the data on an Excel spreadsheet. A score of 50% in each knowledge subsection and skills section of the questionnaire indicated a pass in alignment with the school and university’s grading system [22,25]. Statistical analyses were conducted using IBM Statistical Package for Social Sciences (SPSS) version 26. One-way between-groups ANOVA with planned comparisons was used to compare the effect of traditional and integrated curricula on participants’ knowledge and skills. Participants’ mean scores were compared to the average score of 50% using one-sample *t*-test to determine the pass rate. Linear regression analysis was used to determine the effect of participants’ demographics on knowledge and skills mean scores. Cronbach’s alpha was calculated to quantify the questionnaire’s reliability.

## 3. Results

### 3.1. Participants’ Demographics

A total of 142 final year pharmacy students participated in the study in three different cohorts between 2017 and 2019 based on their exposure to a fragmented or an integrated MCH curriculum. The results in Table 3 showed that 96% of the participants were between 20 and 30 years old, and 69% were female (98). Overall, thirteen participants (9%) were parents, while less than half of the participants (42%) did a locum.

### 3.2. Participants’ Knowledge and Skills Assessment

**Figure 1:**
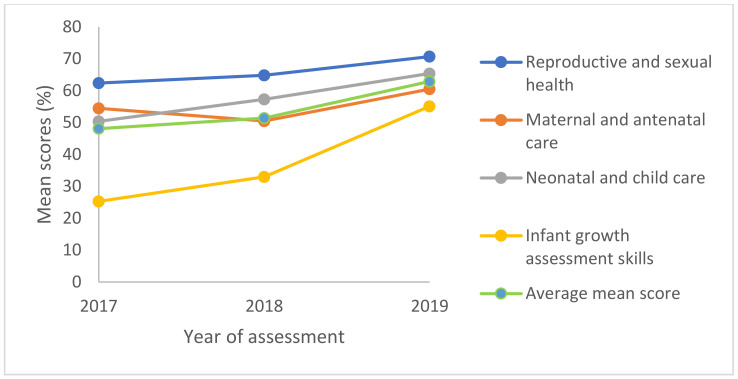
A plot of participants’ mean scores in the three assessments shows that the highest scores were obtained in the 2019 assessment.

As seen in Figure 1, the 2019 cohort obtained the highest mean score (70.7%, SD = 13.6) and scored consistently higher in all the MCH components than the two cohorts exposed to the traditional curriculum. The highest mean scores across the three cohorts were observed in the reproductive and sexual health components, while the lowest was recorded in the infant growth assessment skills components.

In Table 4, participants’ mean scores were compared with the pass mark of 50%. The reproductive and sexual health components remained the strongest for all three cohorts, and the mean scores obtained were significantly (*p* ≤ 0.05) above the pass mark. On the contrary, none of the cohorts scored a significant average pass mark in the infant growth assessment skills components. Overall, a significant average mean score was observed in the 2019 assessment. The Cronbach’s Alpha A test of the reliability of the questionnaire was 0.739.

### 3.3. Comparison of Participants’ Mean Scores in 2019 vs. 2017 and 2018 Assessments

In Table 5, one-way between-groups ANOVA with post hoc test compared participants’ mean scores in the traditional and integrated curricula. The results showed a statistically significant difference in participants’ mean scores between the 2019 and 2018 cohorts in the maternal and antenatal care components (*p* = 0.000, MD = 10). However, significant differences were observed in the mean scores of 2019 and 2017 cohorts in the reproductive and sexual health (*p* = 0.007, MD = 8.3) and neonatal and child care components (*p* = 0.000, MD = 15). Significant differences were observed between the integrated curriculum and traditional curriculum (2018 and 2017) in the infant growth assessment skills components (*p* = 0.000; MD 2018 = 22, MD 2017 = 29.8) and the average mean scores of participants (*p* = 0.000; MD 2018 = 11.5, MD 2017 = 14.8).

No statistically significant differences were observed between the mean scores of participants exposed to the traditional curriculum in 2018 and 2017 in all the MCH components and the average mean score.

### 3.4. Effect of Participants’ Demographic Data on Knowledge and Skills Scores

Linear regression analysis was used to identify the statistically significant effect of participants’ demographic data on knowledge and skills mean scores in the three assessments (Table 6). Participants aged 20–30 years were associated with a lower score of 47% (β = −47.4) in the infant growth assessment skills section than the older students (31–40 years) in the 2017 assessment. In the 2018 assessment, being female was associated with a higher mean score of 11% in maternal and antenatal care than being male.

Similar to the 2017 assessment, participants who were 20–30 years old were associated with lower marks by 32% in maternal and antenatal care (β = −32.6) and 35% in neonatal and child care (β = −35.5) in the 2019 assessment. In addition, the same age range was associated with reducing the average mean score by 27.5% (β = −27.5). The multicollinearity of the demographic variables was not assessed, since that was not the focus of the study. Locum experience and parenting status did not affect participants’ knowledge and skills.

## 4. Discussion

Changing from a traditional to an integrated curriculum was advocated for medical and health care professional education over the years because it is student-centred and can build competence by enhancing learning, knowledge retention, and application [20,33,34]. Koster et al. opined that for a competency-based curriculum to be achieved, competencies such as knowledge, skills, and behaviour relevant to a professional situation such as MCH should be integrated [18]. Zaman and Rauf demonstrated the advantages of integrating the MCH components in a medical curriculum in Pakistan [20]. Egieyeh et al. arrived at the same conclusion in a similar study of a pharmacy curriculum in South Africa [22]. This study further highlighted the knowledge and skills gains of an integrated MCH pharmacy curriculum over a traditional one through comparative evaluation between different cohorts, which was the first accomplished in South Africa.

### 4.1. Participants’ Knowledge and Skills Assessment

Although statistical significance was not observed in participants’ mean scores in all the MCH components between the integrated and traditional curriculum (2018 and 2017), the mean scores recorded for the 2019 cohort were higher than that of the 2018 and 2017 cohorts. The 2019 cohort also had the highest mean differences and significant average mean score, which showed that integrating the MCH contents in the curriculum enhanced the knowledge and skills of the 2019 final year pharmacy students. Tsinopoulos et al. reported a similar result. The overall mean score of students exposed to an integrated curriculum in ophthalmology at a Greek medical school was higher than that of students exposed to a traditional teaching method [35]. A statistically significant difference in participants’ mean scores was reported for one MCH component in the 2019 and 2018 cohorts. This may be attributed to improved teaching methods in 2018 compared to 2017. However, there was no significant difference in participants’ performance between 2017 and 2018 in the traditional curriculum, indicating that the curriculum integration enhanced participants’ performance.

The effect of longitudinal dispersion of curriculum content was highlighted in this study, as the result showed that the reproductive and sexual health component consistently recorded the highest mean score in each assessment, even in the traditional exposure. Competency was developed as students progressed through the curriculum because the content was taught across students’ years of study and the product demonstrated practical and experiential learning activities [18,22]. This provides evidence for the readiness of pharmacy graduates to provide reproductive and sexual health care services as part of the preventive drivers of MCM [28]. The SAPC requires pharmacists to undergo and register appropriate supplementary training to provide comprehensive reproductive health services. However, the competencies gained from the undergraduate training would enable them to engage in multidisciplinary teams and conduct health education programmes about family planning options. These are important not only for first pregnancies but also to decrease the high rate of repeat pregnancies in adolescents [10,30,36,37,38]. In addition, active learning methods such as small group seminars in place of lectures, role plays, journal club presentations and practical workshops that received positive feedback from students in other curriculum integration studies may be adopted [28,35].

### 4.2. Effect of Participants’ Demographic Data on Knowledge and Skills Scores

The effect of the integrated MCH curriculum may be weakened in this study, as most of the participants (96%) who were between 20 and 30 years old were associated with lower scores in maternal and antenatal care, neonatal and child care, and consequently, the average mean score. This may be attributed to the low number of participants with children (2%) since most parents would be eager to learn and retain MCH knowledge and skills, as research has shown that parental knowledge influences children’s developmental outcomes [39].

### 4.3. Limitations and Recommendations

There are limitations to our study. Data collection was from one cohort in the integrated curriculum, while it was collected from two cohorts in the traditional curriculum. The synergistic effect of the two-year data may have reduced the difference observed between the two curriculum exposures. Since a convenient sampling method was used in the three assessments, the results may not be a true reflection of the competencies of the entire class. In addition, the 2019 cohort had two prior exposures to the questionnaire in their second-year pre and post-exposure assessments. The repeated exposures may have given them an edge over the 2017 and 2018 cohorts who completed the questionnaire once in their final year of study. As the assessments did not count for course grades, the participants’ stakes were low, which may have affected their performance. Infant growth assessment skills were examined as a written assessment rather than the traditional objective structured clinical examination or practical evaluation due to inadequate manpower. The assessment process may undermine the results of that section.

Clinical nurse practitioners traditionally offer MCH at the primary care level. Increased multidisciplinary training would develop the competencies of entry-level graduates towards team-based care for South Africa’s decentralised district-based primary care, which is crucial for health system strengthening [40].

Further research to assess practising pharmacists’ MCH knowledge, attitude, and practices in South Africa should be carried out to inform the introduction of a continuing professional development programme.

## 5. Conclusions

The integration of the MCH content in the curriculum enhanced students’ knowledge significantly. This supports the clamour for the training of health care professionals who are fit for purpose and able to meet the healthcare needs of the community they serve. To ensure adequate knowledge and skills acquisition and retention, active learning strategies, longitudinal dispersion of curriculum content across a student’s years of study, interprofessional collaboration and avenues for work-based learning are vital.

## Figures and Tables

**Table 1:** MCH framework indicating the traditional and integrated content per year of study aligned with three SAPC domains and competencies.

	Traditional MCH Curriculum Content for the 2017/2018 Cohort			Integrated MCH Curriculum Content for the 2019 Cohort				
Discipline/Module Code	Semester	Lecture component/ Duration	Skills/SliP ^2^ Activity/Duration	SAPC ^6^ Domain	Competencies	Semester	Lecture Component/Duration	Skills/SLiP Activity/ Duration
Introduction to Pharmacology and Clinical Pharmacy (PHC 123)	BPharm ^1^ 1 Semester 2 (2014/2015)	Environmental and nutritional health (diarrhoeal disease, deworming, Vitamin A supplementation) (3 h)	SLiP Environmental health visit to an underserved community (8 h) ORS ^3^ salt and sugar dry powder preparation on campus (4 h)	Public health	Promotion of health and wellness, medicines information, professional and health advocacy, primary health care	BPharm 1 (2016)	The relevant module content was not integrated into MCH until 2018.	
Pharmacology and Clinical Pharmacy (PHC 213)				Safe and rational use of medicines and medical devices	Patient consultation, patient counselling, medicines, and medical devices safety, pharmacist-initiated therapy, pharmacovigilance	BPharm 2 Semester 1 (2017)		Infant growth assessment skills practical on campus (8 h)
Pharmacology and Clinical Pharmacy (PHC 223)	BPharm 2 Semester 2 (2015/2016)	Pregnancy care, infant care communicable diseases, immunisation (2 h crash course), contraception part 1 (2 h)	Contraceptive products practical demonstration (2 h) SLiP-MCH ^4^ programme at a PH5 ^5^ facility (9 h)			BPharm 2 Semester 2 (2017)	Pre-pregnancy and antenatal care, pregnancy care, infant care, communicable diseases of childhood, immunisation against diseases of childhood (EPI ^7^), contraception part 1 (15 h)	Contraceptive products practical demonstration (2 h) SLiP-MCH programme at a PHC facility (9 h)
Pharmacy Practice (PPR 324)				Professional and personal practice	Patient-centred care, decision making, collaborative practice, communication	BPharm 3 Semester 2 (2018)		Externship programme at a retail pharmacy or PHC facility (48 h)
Pharmacology and Clinical Pharmacy (PHC323)	BPharm 3 Semester 2 (2016/2017)	Reproductive hormones (2 h)					Participants were exposed to the relevant module content although it was not integrated.	
Pharmacy Practice (PPR 414)	BPharm 4 Semester 1 (2017/2018)	Contraception part 2 (2 h)		Safe and rational use of medicines and medical devices	Patient consultation, patient counselling, medicines, and medical devices safety, pharmacist-initiated therapy, pharmacovigilance	BPharm 4 Semester 1 (2019)	Contraception part 2 (2 h)	

^1^ Bachelor of Pharmacy, ^2^ Service-learning in Pharmacy, ^3^ Oral rehydration solution^, 4^ Service-learning in pharmacy: maternal and child health, ^5^ Primary health care, ^6^ South African Pharmacy Council, ^7^ Expanded Programme on Immunisation.

**Table 2:** The integrated MCH framework content and the associated assessment tools.

Year of Study	Lecture Topic/Activity	Assessment Tools
BPharm 1	Environmental and nutritional health lectures	Quiz, test, final exam
	SLiP ^1^ Environmental health visit to an underserved community	Reflection report writing, supervised ORS ^2^ dry powder preparation
BPharm 2 Semester 1	Infant growth assessment skills practical on campus	Group work on assigned cases
BPharm 2 Semester 2	Pre-pregnancy and antenatal care, pregnancy care, infant care Communicable diseases of childhood, immunisation, contraception part 1	Quiz, test, final exam
	SLiP-MCH ^3^ programme at a primary health care facility	Quiz, reflection report writing
	Contraceptive products practical demonstration	Group work on assigned cases
BPharm 3 Semester 2	Externship programme in a retail pharmacy/health care facility	Reflection report writing
BPharm 4 Semester 1	Contraception part 2	Quiz, test, final exam

^1^ Service-learning in Pharmacy, ^2^ Oral rehydration solution, ^3^ Service-learning in pharmacy-maternal and child health.

**Table 3:** Demographic data of study participants for the 2017, 2018 and 2019 cohorts.

	2017 *n* = 54 (%)	2018 *n* = 41 (%)	2019 *n* = 47 (%)	Total *n* = 142 (%)
Age (in years)				
20 to 30	53 (98)	39 (95)	44 (94)	136 (96)
31 to 40	1 (2)	2 (5)	3 (6)	6 (4)
Gender				
Female	34 (63)	28 (68)	36 (77)	98 (69)
Male	20 (37)	13 (32)	11 (23)	44 (31)
Parenting status				
Have children	8 (15)	4 (10)	1 (2)	13 (9)
No children	46 (85)	37 (90)	46 (98)	129 (91)
Locum experience (in years)				
None	22 (41)	25 (61)	35(74)	82 (58)
1 to 2	26 (48)	16 (39)	11 (24)	53 (37)
>2	6 (11)	-	1(2)	7 (5)

**Table 4:** Participants’ mean scores in the three assessments in percentage compared to the university’s stipulated pass mark of 50%.

	2017 Cohort (*n* = 54)	2018 Cohort (*n* = 41)	2019 Cohort (*n* = 47)
MCH Component	Mean (SD) ^1^	Mean (SD)	Mean (SD)
	MD ^2^, *p*-value ^3^	MD, *p*-value	MD, *p*-value
Reproductive and sexual health	62.4 (15.9)	64.8 (15.5)	70.7 (13.6)
	12.4, 0.000	14.8, 0.000	20.7, 0.000
Maternal and antenatal care	54.5 (17.8)	50.5 (16.4)	60.5 (18.8)
	4.5, 0.07	0.5, 0.8	10.5, 0.000
Neonatal and child care	50.4 (20.6)	57.3 (18.8)	65.4 (18.6)
	0.4, 0.88	7.3, 0.000	15.4, 0.000
Infant growth assessment skills	25.3 (20.0)	33.0 (24.9)	55.1 (25.9)
	−24.7, 0.000	−17.0, 0.000	5.1, 0.2
Average mean score	48.1 (14.5)	51.4 (13.9)	62.9 (13.7)
	−1.9, 0.35	1.4, 0.5	12.9, 0.000
Cronbach’s Alpha A test (*n* = 4) ^4^	0.739		

^1^ Standard deviation, ^2^ Mean Difference—the difference between the mean scores obtained in the assessment and the university’s stipulated pass mark of 50%, ^3^ Significant at *p* ≤ 0.05, ^4^ Number of items analysed.

**Table 5:** Comparing participants’ mean scores in the three assessments.

MCH Component	Year Compared	Mean Difference (MD) ^1^ %	*p*-Value ^2^
Reproductive and sexual health	2019 to 2018	5.9	0.069
	2019 to 2017	8.3	0.007
	2018 to 2017	2.4	0.454
Maternal and antenatal care	2019 to 2018	10.0	0.009
	2019 to 2017	6.1	0.089
	2018 to 2017	−4.0	0.283
Neonatal and child care	2019 to 2018	8.1	0.054
	2019 to 2017	15.0	0.000
	2018 to 2017	6.9	0.088
Infant growth assessment skills	2019 to 2018	22.0	0.000
	2019 to 2017	29.8	0.000
	2018 to 2017	6.9	0.088
Average mean scores	2019 to 2018	11.5	0.000
	2019 to 2017	14.8	0.000
	2018 to 2017	3.3	0.265

^1^ Mean Difference is the difference between the mean scores obtained in two assessments, ^2^ Significant at ≤ 0.05.

**Table 6:** Effects of participants’ demographic variables on knowledge and skills mean scores.

Year of Assessment	MCH Component	Variable	Unstandardised Coefficients		
			B ^1^	Standard error	*p*-value ^2^
2017	Infant growth assessment skills	(Constant)	63.7	20.9	0.004
		Age (20–30 years)	−47.4	20.6	0.030
2018	Maternal and antenatal care	(Constant)	78.8	13.4	0.000
		Female	11.0	5.1	0.04
	Average total score	(Constant)	68.6	11.9	0.000
		Female	9.2	4.5	0.049
2019	Maternal and antenatal care	(Constant)	61.8	19.2	0.003
		Age (20–30 years)	−32.6	13.3	0.019
	Neonatal and child care	(Constant)	72.1	18.4	0.000
		Age (20–30 years)	−35.5	12.8	0.008
	Average total score	(Constant)	78.7	13.2	0.000
		Age (20–30 years)	−27.5	9.2	0.004

^1^ B column contains the unstandardised beta coefficients that depict the magnitude and direction of the effect on the outcome variable^.^^2^ *p*-value ≤ 0.05; then, that variable has a significant association with the outcome variable.

## Data Availability

The data presented in this study are available on request from the corresponding author. The data are not publicly available due to ethical reasons.

## Supplementary Materials

The following supporting information can be downloaded at: https://www.mdpi.com/article/10.3390/pharmacy10030062/s1, Questionnaire file.
