# Supplementary material for: Comparative Evaluation of Pharmacy Students’ Knowledge and Skills in Maternal and Child Health: Traditional versus Integrated Curriculum
Source: Pharmacy (Basel). 2022 Jun 7;10(3):62. doi: 10.3390/pharmacy10030062 (PMC9231310; doi:10.3390/pharmacy10030062)
Supplement: Supplementary file 1 [file pharmacy-10-00062-s001.zip › pharmacy-1705831-Supplementary.pdf]

FACULTY OF NATURAL SCIENCES
Private Bag X17 Bellville 7535
Telephone +27 21 9592190
Fax +27 21 9593407

APPENDIX 2

CONSENT FORM

Research title: Comparative Evaluation of Students' Knowledge and Skills in Maternal and Child Health: Traditional versus Integrated Curriculum

I confirm that I have read and understand the information sheet for the above study. I have had the opportunity to consider the information, ask questions and have had these answered satisfactorily.

1. I understand that my participation is voluntary and that I am free to withdraw at any time without giving any reason.
2. I understand that the study will not affect my legal rights and the data collected will not be linked to me in any way and full confidentiality is guaranteed
3. It has been explained clearly to me that the study was approved by relevant ethics committee
4. I agree to take part in the above study.

____________________	____________________	____________________
Participant Unique ID	Date	Signature of participant
____________________	____________________	____________________
Name of researcher	Date	Signature of researcher

UNIVERSITY of the WESTERN CAPE

School of PHARMACY

QUESTIONNAIRE

APPENDIX 1

Research title: Comparative Evaluation of Students' Knowledge and Skills in Maternal and Child Health: Traditional versus Integrated Curriculum

Unique ID:____________________

A. PERSONAL INFORMATION / DEMOGRAPHICS (check as required)

1. AGE:

☐ 20 - 30 years

☐ 31 – 40 years

☐ 41-50 years

2. GENDER:

☐ Male

☐ Female

3. PARENTAL STATUS:

☐ Children

☐ No children

4. LOCUMING :

☐ Yes

☐ No

1. IF YES, HOW LONG:

☐ 1-2 years

☐ > 3 years

B. KNOWLEDGE SECTION *(All questions are allocated one mark except otherwise indicated)*

B.1 REPRODUCTIVE/SEXUAL HEALTH (Circle the correct answer or “I don’t know” if you are not sure of the correct answer. Also, fill the blank spaces with the correct answer or “I don’t know” if you are not sure of the correct answer)

1. Which hormones are present in combined oral contraceptives?
   a. Eostrogen and progestogen
   b. Testosterone and oxytocin
   c. Prolactin and thyroxin
   d. Prolactin and oxytocin
   e. I don’t know
2. Combined oral contraceptives should be started on which of the following days?
   a. Between day 1 and day 5 of the cycle
   b. At any time in the absence of pregnancy
   c. After day 5 of the cycle with additional precaution (barrier method) until 7 hormonal pills have been taken
   d. All of the above
   e. I don’t know
3. Which of the following contraceptive methods’ effectiveness relies on the client’s ability to use them correctly?
   a. Vasectomy
   b. Condoms
   c. IUD
   d. Sub-dermal implant
   e. I don’t know
4. Long-acting reversible contraceptives (LARC) are defined as methods that require administration once in months or cycles. Which of the methods listed below fall within this definition?
   i. Copper IUD
   ii. Levonorgestrel releasing intrauterine system
   iii. Progestogen-only injectables
   iv. Sub-dermal progestogen implants
      a. All of the above
      b. None of the above
      c. ii and iii
      d. i and ii
      e. I don’t know
5. Emergency contraception is absolutely indicated after sexual intercourse in all of the following situations except which one?
   a. One pill is forgotten or 3 hours late with sexual intercourse in the past 5 days
   b. Two pills forgotten during the first 7 active pills
   c. > 2 weeks late for Progestogen-only injections
   d. <2 weeks late for Progestogen-only injections
   e. I don’t know

6. The following information are important before emergency contraception is dispensed except which one?
    a. Date of the last menstrual period
    b. <120 hours since the last episode of unprotected intercourse
    c. How many hours of rest observed
    d. Exclusion of pregnancy
    e. I don't know
7. Dual contraception is encouraged to prevent the transmission of ____________________
8. Oral progestogen-only pills are
    a. Preferred postpartum during lactation
    b. In women over 35 years who smoke and have increased risk of cardiovascular disease
    c. Disrupt the menstrual cycle
    d. All of the above
    e. I don't know
9. Rifampicin, Lopinavir/Ritonavir, Nevirapine are enzyme inducers that interact with oral contraceptives to
    a. Reduce contraceptive effect
    b. Increase contraceptive effect
    c. Stabilize contraceptive effect
    d. Terminate contraceptive effect
    e. I don't know

B.2 MATERNAL/ANTENATAL CARE (Circle the correct answer or "I don't know" if you are not sure of the correct answer. Also, fill the blank spaces with the correct answer or "I don't know" if you are not sure of the correct answer)

1. All non-pregnant women of reproductive age should be advised to commence periconceptional folic acid supplementation (women planning pregnancy). True or false or I don't know?
2. Every pregnant woman should have at least ____ antenatal clinic visits.
    a. 2
    b. 4
    c. 6
    d. 8
    e. I don't know
3. State 4 lifestyle modifications required by a pregnant woman for a healthy pregnancy and baby? (4 marks)

    ____
    ____
    ____
    ____

4. State 4 signs that indicate a pregnancy is in danger. (4 marks)

    ____
    ____
    ____
    ____
    ____

5. When is ARV therapy initiated in newly diagnosed HIV positive pregnant women?
    a. Immediately HIV status is confirmed
    b. CD4 count <500 cells/mm^3
    c. Viral load <1000 IU/ml
    d. Immediately after birth
    e. I don't know
6. Which of the following is non-teratogenic?
    a. ACE inhibitors
    b. Vitamin A derivatives
    c. Statins
    d. None of the above
    e. I don't know
7. Which of these factors influence the manifestation and severity of teratogenicity?
    a. Gestation period
    b. Dose and duration of therapy
    c. Degree of drug transfer across the placenta
    d. All of the above
    e. I don't know

Please answer questions 17, 18 and 19 in the table below according to the instruction at the top of the table. (4 marks)

Complaint	State one cause in the blank cell.	Give one Non-pharmacological treatment/prevention
8. Morning sickness	Reduced gastric motility/high hormonal levels	
9. Heartburn		
10. Vaginal thrush	Alteration in pH balance/Candida albicans	

UNIVERSITY of the WESTERN CAPE

School of PHARMACY

B.3 NEONATAL AND CHILD CARE (Circle the correct answer or "I don't know" if you are not sure of the correct answer. Also, fill the blank spaces with the correct answer or "I don't know" if you are not sure of the correct answer)

1. Exclusive breastfeeding (EBF) is defined as giving only breast milk to infants for the first ______ of life.
   a. 2 months
   b. 4 months
   c. 6 months
   d. 12 months
   e. I don't know
2. An HIV exposed infant is one whose mother is HIV infected or whose HIV infection has not been confirmed or excluded. Which ARV medication is given to such infants at birth?
   ____
3. WHO recommends that HIV positive women who are on ART should exclusively breastfeed their babies. True or false?
4. Cracked nipples during breastfeeding is a result of which of the following?
   i. Poor positioning of the baby to the nipple
   ii. Incorrect attachment to the nipple
   iii. Removing the baby from the breast before suction is broken
   iv. Breastfeeding the baby while lying down
   a. All of the above
   b. i, ii, iii
   c. i and ii
   d. Ii and iii
   e. I don't know
5. The Expanded Program on Immunization (EPI) covers the major killer diseases of infanthood. List three of such diseases. (3 marks)
   ____
   ____
   ____
6. How is diarrhoea treated in infants and children?
   ____
   ____
7. Deworming agents are initially given to children at ______________, subsequently every ______.
   a. 6 months, 6 months
   b. 6 months, 12 months
   c. 12 months, 6 months
   d. 12 months, 12 months
   e. I don't know
8. Outline one pharmacological and non-pharmacological treatment ***for diaper/nappy rash*** (2 marks)
   Pharmacological:____
   Non-pharmcological:____
   ____

9. Which of the following can be used in the treatment of mastitis?
    a. Apply warm compresses
    b. Drink plenty of clear fluids
    c. Panado
    d. Antibiotics
    e. All of the above
    f. I don't know

C. SKILLS ASSESSMENT (Please give a brief explanation to each of the following questions or "I don't know" if you are not sure of the correct answer)

1. Please explain how an infant's (0-12 months) height is measured? (2 marks)

____
____
____

2. Should an infant be fully clothed or undressed during weight measurement? (1 mark)

____

3. How is an infant's head circumference measured? (2 marks)

____
____
____

4. Why is an infant's head circumference measured? (1 mark)

____
____
____

5. What is MUAC? (1 mark)

____
____
____

6. Why is MUAC measured? (1 mark)

____
____
____
____

*** THE END. THANK YOU!!***
